# COVID-19 and the burning issue of drug interaction: never forget the ECG

**DOI:** 10.1136/postgradmedj-2020-138093

**Published:** 2020-08-20

**Authors:** Carlotta Sciaccaluga, Matteo Cameli, Daniele Menci, Giulia Elena Mandoli, Nicolò Sisti, Paolo Cameli, Federico Franchi, Sergio Mondillo, Serafina Valente

**Affiliations:** Department of Medical Biotechnologies, Section of Cardiology, University of Siena, Siena, Italy; Department of Medical Biotechnologies, Section of Cardiology, University of Siena, Siena, Italy; Department of Medical Biotechnologies, Section of Cardiology, University of Siena, Siena, Italy; Department of Medical Biotechnologies, Section of Cardiology, University of Siena, Siena, Italy; Department of Medical Biotechnologies, Section of Cardiology, University of Siena, Siena, Italy; Respiratory Diseases and Lung Transplantation, Department of Medicine, Surgery and Neurosciences, Siena University Hospital, Siena, Italy; Department of Medicine, Surgery and Neuroscience, Anesthesia and Intensive Care Unit, University of Siena, Siena, Italy; Department of Medical Biotechnologies, Section of Cardiology, University of Siena, Siena, Italy; Department of Medical Biotechnologies, Section of Cardiology, University of Siena, Siena, Italy

**Keywords:** Cardiology, adult cardiology, infectious diseases, public health

## Abstract

The coronavirus disease of 2019 (COVID-19), caused by severe acute respiratory syndrome coronavirus 2 (SARS-CoV2), has been rapidly escalating, becoming a relevant threat to global health. Being a recent virus outbreak, there are still no available therapeutic regimens that have been approved in large randomised trials and so patients are currently being treated with multiple drugs. This raises concerns regarding drug interaction and their implication in arrhythmic burden. In fact, two of the actually used drugs against SARS-CoV2, such as chloroquine and the combination lopinavir/ritonavir, might determine a QT (the time from the start of the Q wave to the end of the T wave) interval prolongation and they show several interactions with antiarrhythmic drugs and antipsychotic medications, making them prone to an increased risk of developing arrhythmias. This brief review focuses the attention on the most relevant drug interactions involving the currently used COVID-19 medications and their possible association with cardiac rhythm disorders, taking into account also pre-existing condition and precipitating factors that might additionally increase this risk. Furthermore, based on the available evidence and based on the knowledge of drug interaction, we propose a quick and simple algorithm that might help both cardiologists and non-cardiologists in the management of the arrhythmic risk before and during the treatment with the specific drugs used against SARS-CoV2.

## BACKGROUND

The coronavirus disease of 2019 (COVID-19), caused by severe acute respiratory syndrome coronavirus 2 (SARS-CoV2), has rapidly become a relevant threat to health as well as to economy in the whole world.^1 2^ The infection has been dramatically escalating over a relatively short period of time, forcing the WHO to declare it a pandemic.

**Figure 1:**
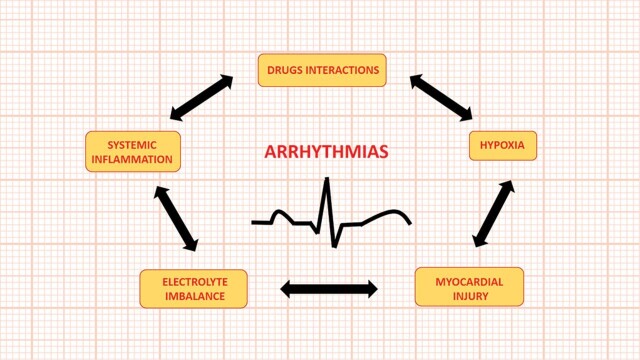
Mechanisms of the onset of ventricular arrhythmias in patients with coronavirus disease of 2019.

An interesting aspect to take into consideration is that the development of arrhythmias is a relatively common manifestation reported in patients affected by COVID-19,^3^ with a higher prevalence among those admitted to the intensive cardiac unit.^4^ Published data on the type of arrhythmias and the timing of the onset are not available yet. Several are the possible involved underlying mechanisms, ranging from systemic inflammation, myocardial injury, electrolyte imbalance to hypoxia and drug interactions (figure 1). In particular, patients are currently being treated with several drugs, since there are still no available therapeutic regimens that have been approved in large randomised trials. According to the first Chinese reports,^5–7^ the majority of patients with COVID-19 received both antiviral and antibiotic agents. Furthermore, great attention has been recently paid to immune-modulating medications, already used in several rheumatologic diseases, such as chloroquine, hydroxychloroquine and tocilizumab.^1^ However, it has to be stressed that the combination of these agents, all of which are characterised by own side effects, might increase the likelihood of cardiac rhythm disorders, especially in case of concomitant use of pro-arrhythmic drugs, such as antiarrhythmic and antipsychotic drugs. These medications might be responsible for arrhythmias due to different mechanisms. First, as a direct effect, it can be elicited on myocardial repolarisation, inducing QT interval prolongation, especially in the presence of electrolyte imbalance. Moreover, drug interactions at different levels (absorption, carriage by plasmatic proteins, hepatic metabolism, renal excretion) might raise plasma concentration of a certain drug metabolite. This brief review focuses on the main pharmacological interactions.

The figure shows the main mechanisms upon which arrhythmias might develop in patients with COVID-19. One of the main causes is determined by drug interactions, which therefore have to be carefully considered by the clinician, looking for signs that indicate a higher risk patient.

### Chloroquine and hydroxychloroquine

Chloroquine and hydroxychloroquine are two aminoquinolines used as antimalaria agents. Even in previous virus outbreaks, they showed antiviral effects, and they are currently showing promising results in treating SARS-CoV2 infection.^8^ Their use in patients with concomitant CVD (cardiovascular diseases) has to take into consideration not only a possible direct myocardial toxicity but also drug interactions that can enhance the side effects on the cardiac conduction system and therefore on the cardiac rhythm. Chloroquine use may increase depolarisation length duration and Purkinje fibre refractory period ultimately leading to atrioventricular nodal or infranodal delay. Both drugs are accumulated in lysosomes where they increase pH and can induce protein inactivity. This property seems to be linked to the increased burden of atrial and ventricular arrhythmias during administration and to the risk of fascicular block.^9^ Both of these agents can induce QT interval prolongation,^10^ especially with chronic use, even though the use of hydroxychloroquine is reported as a rare cause of this condition.^11^ This effect is not well understood but Capel *et al* demonstrated in an animal model an inhibitory effect of the hydroxychloroquine on the hyperpolarisation-activated current ion channels, delayed rectifier potassium currents and L-type calcium ion currents.^12^ However, as mentioned above, their combination with antiviral agents, antibiotics and antiarrhythmic drugs increases this risk. In particular, a recent evidence has suggested that combining hydroxychloroquine with azithromycin boosts the overall antiviral effect.^13^ In this context, it is essential to keep in mind that it has been well recognised how this macrolide agent is able to determine a QT interval prolongation, especially in older patients,^14^ whereas this effect might be negligible in children.^15^

### Antiviral agents

The currently used antiviral drugs against SARS-CoV2 infection include lopinavir/ritonavir, a combination of protease inhibitors, remdesivir and ribavirine, which both exert a broad-spectrum antiviral activity.^3^ The risk of QT interval prolongation has been documented in vitro with the association of lopinavir/ritonavir and it seems to be induced by the inhibition of human ether-a-go-go-related gene (hERG) current. However, a discrepancy exists between in vitro and in vivo results and has led to requirement of clinical QT corrected (QTc) assessment even in the absence of a clear demonstration of prolonged repolarisation. Theoretical models suggest that the degree of hERG blockade given by lopinavir/ritonavir is expected to produce a prolongation of QTc interval of <5 ms.^16^ High-grade atrio-ventricular blocks are additional side effects, and multiple drug interactions are possible due to the inhibition of CYP3A. In particular, it has been documented that this combination increases the plasma concentration of both amiodarone and dronedarone.^3^ Lastly, lopinavir/ritonavir might lead to augmented plasma level of digoxin through p-glycoprotein inhibition.^3^

Remdesivir is an antiviral agent that has been shown to inhibit the COVID-19,^17^ and it is currently under clinical trials, as ribavirin is. To our knowledge, there is no current data on the risk of developing arrhythmias induced by these two drugs.

### Interleukin (IL)-6 receptor inhibitors

Both tocilizumab and sarilumab are IL-6 receptor inhibitor and both medications have been approved for ongoing clinical trials. Tocilizumab is usually used for rheumatoid arthritis and cytokine release syndrome.^2^ There are ongoing clinical trials^18–20^ investigating its role as a treatment of severe form of COVID-19 infection. In literature, there are no reported effects of tocilizumab on QT interval in healthy subjects, both considering therapeutic and sovra-therapeutic doses.^21^ Furthermore, some studies suggest a positive effect of tocilizumab in reducing QT interval probably through a decrease in C reactive protein, in patients affected by rheumatoid arthritis.^22^ Similarly, sarilumab is used to treat adult patients with moderately to severely active rheumatoid arthritis who have had an inadequate response or intolerance to other therapies.^23^ Through the inhibition of IL-6 receptor, the use of both sarilumab and tocilizumab might restore the activity of cytochrome P450s, which are proven to be downregulated by infection and inflammation.^24^ This mechanism should be taken into consideration when these drugs are used in combination with other therapies that are metabolised by cytochrome P450s.

### Other drugs

Anakinra is a recombinant IL-1 receptor antagonist currently approved for the treatment of rheumatoid arthritis. A small retrospective cohort study carried on in Italy showed that this drug could increase the survival of patients with COVID-19 and improve respiratory status.^25 26^ In a murine model studied by De Jesus *et al*, anakinra improved conduction velocity, reduced action potential duration dispersion, improved intracellular Ca^2+^ handling, decreased transmembrane potential, and reduced spontaneous and inducible ventricular arrhythmias.^27^ Also, lithium might be effective against COVID-19, inhibiting apoptosis and glycogen synthase kinase-3 beta which are involved in inflammatory pathways.^28^ Lithium determines electrical changes dependent on both duration of treatment and the serum drug level, and usually manifests as sinus node dysfunction, sinoatrial blocks, PR (the time from the beginning of the P wave until the beginning of the QRS complex) prolongation, QT prolongation/dispersion and ventricular tachyarrhythmias.

## CAREFUL CONSIDERATION OF PRECIPITATING FACTORS

As mentioned above, the genesis of arrhythmias in COVID-19 is multifactorial, and therefore all the precipitating factors must be addressed and considered when specific COVID-19 treatment is started. Table 1 summarises the most commonly encountered precipitating factors in these patients. First of all, all modifiable factors must be corrected and frequently rechecked during this treatment: electrolyte imbalances and careful monitoring of diuresis and diuretic therapy are the most relevant. Second, liver or renal dysfunction, which is relatively common in critically ill patients, must be taken into consideration to properly adjust the dose of drugs according to metabolism and clearance, avoiding dangerous overdosage. Acute myocardial infarction, myocarditis and in general situations in which the myocardium suffers an insult should be also considered in the risk stratification of arrhythmias, since they are well-known conditions that might predispose to the onset of cardiac rhythm disorders.^29^ In the overall risk estimation, two emerging concepts should be assessed, hypoxia and high grade of systemic inflammation.^30 31^ For instance, several studies have suggested that cytokines might be involved in arrhythmogenesis, with several mechanisms, ranging from triggered activity to creation of reentrant loops.^32 33^ On the other hand, a recent investigation showed that acute cardiac hypoxia induces late sodium current in the heart, leading to potentially life-threatening arrhythmias.^31^ These elements might characterise patients with COVID-19 experiencing aggressive forms of the infection, and since they are the ones that mostly necessitate aggressive therapies, signs of increased arrhythmogenic risk should be frequently assessed and possibly managed.

**Table 1:** Precipitating factors that might contribute to the onset of arrhythmias

Precipitating factors	
Hypoxia	Pneumonia Acute respiratory distress syndrome Prone positioning (transient, only during the initial phase)
**Systemic inflammation**	
Hydroelectrolytic imbalance	Diuretics Hypovolemia Renal failure
Acute myocardial injury	Myocardial infarction Myocarditis Sepsis Acute respiratory distress syndrome Acute renal failure Pulmonary embolism Cardiotoxicity
**Heart failure**	

## CLINICAL MANAGEMENT AND CONCLUSIONS

Drug interactions and their correlated arrhythmogenic risk are relevant and current problems for patients with COVID-19. There is no actual therapy whose efficacy has been tested in a large randomised trial, and therefore these patients are being treated with those multiple agents that have shown some positive results. As a consequence, it is important to carefully consider the possible effects of these agents, combined with other concomitant factors, in order to avoid as much as possible the onset of malignant arrhythmias that might contribute to raise the mortality rate of these patients. As mentioned above, the clinician should pay particular attention to antiarrhythmic drugs, sedatives such as propofol and antipsychotic medications, which are commonly used for delirium treatment. Table 2 summarises the main interaction of the currently used COVID-19 drugs that increase the risk of developing cardiac rhythm disorders.

**Table 2:** Relevant pro-arrhythmic drug interactions involving the most commonly used COVID-19 medications.^34^

COVID-19 drugs	Cardiovascular effects	Drugs to be avoided
Chloroquine (hydroxychloroquine)	- QT prologation - Cardiotoxicity	Amiodarone Flecainide Mexiletine Ziprasidone
	Drugs to use with caution	
	*Anti-COVID-19 drugs*: Lopinavir/ritonavir	*Anaesthetics*: Propofol Sevoflurane
	*Antiarrhythmics*: Digoxine Disopyramide Dofetilide Ivabradine Metoprolol Nebivolol Propafenone Propranolol Quinidine Ranolazine Timolol	*Anticonvulsivants*: Chlorpromazine Clozapine Haloperidol Iloperidone Lovomepromazine Fluphenazine Quetiapine Perphenazine Pimozide Risperidone Sulpride Thioridazine Tiapride Zotepine Zuclopenthixol
	*Antimicrobials*: Azithromycin Clarithromycin Erythromycin Levofloxacin Ofloxacin Moxifloxacin	*Antidepressants*: Citalopram Escitalopram Lithium
Lopinavir/ritonavir	- AV (atrio-ventricular) block - QT prolongation - Increased amiodarone and dronedarone plasma concentration	Amiodarone Disopyramide Dofetilide Flecainide Ivabradine Ranolazine Lercanidipine Sildenafil Pimozide Quetiapine Ziprasidone Midazolam Triazolam
	Drugs to use with caution	
	*Anti-COVID19 drugs*: Chloriquine/hydroxychloroquine	*Antidepressants*: Citalopram Escitalopram Lithium
	*Lipid-lowering agents*: Atorvastatina Lovastatina Rosuvastatina	*Steroids and immunosuppressants*: Sirolimus Tacrolimus Ciclosporin
	*Antiarrhythmics*: Lidocaine Quinidine Mexiletine Propafenone Digoxine Atenolol Bisoprolol Carvedilol Metoprolol Nebivolol Oxprenolol Pindolol Propranolol Timolol Amlodipine Diltiazem Verapamil Nicardipine Nifedipine Labetalol	*Anticonvulsivants*: Chlorpromazine Clozapine Haloperidol Iloperidone Lovomepromazine Fluphenazine Perphenazine Pimozide Risperidone Sulpride Thioridazine Tiapride Zotepine Zuclopenthixol
Remdesivir	Cardiovascular effects	Drugs to be avoided
	No known proven effects	//
	Drugs to use with caution	
	//	
Favipiravir *	Cardiovascular effects	Drugs to be avoided
	No known proven effects	//
	Drugs to use with caution	
	//	
Tocilizumab	Cardiovascular effects	Drugs to be avoided
	Fluid retention	//
	Drugs to use with caution	
	Drugs that are CYP450 substrates	
Sarilumab	Cardiovascular effects	Drugs to be avoided
	Increased triglycerides and low-density lipoprotein-cholesterol	//
	Drugs to use with caution	
	Drugs that are CYP450 substrates	
Anakinra	Cardiovascular effects	Drugs to be avoided
	No known proven effects	//
	Drugs to use with caution	
	//	

*This drug is currently not approved in USA and UE. COVID-19, coronavirus disease of 2019; CYP450, cytochrome P450.

**Figure 2:**
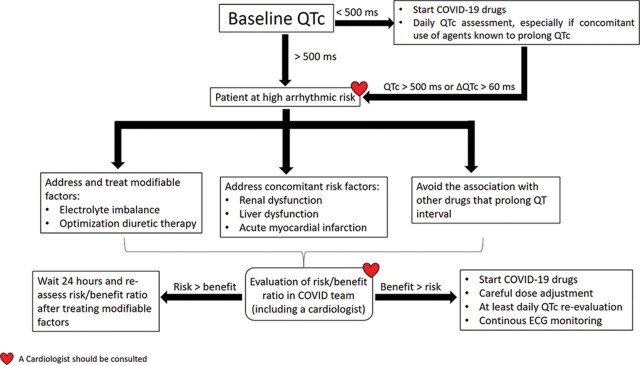
Management of QT corrected (QTc) interval prolongation in patients with coronavirus disease of 2019 (COVID-19).

Based on the available information, an ECG with QTc interval measurement should be performed in all patients with COVID-19, in order to have a baseline value. Furthermore, in those where specific treatment is warranted, it would be important to assess the individual arrhythmic risk profile, addressing and treating possible modifiable factors, such as electrolyte imbalance, fever and diuretic therapy. In addition to that, careful dose adjustment should be made according to the patient’s hepatic and renal function. In line with a recent focus,^10^ a baseline QTc interval above 500 ms or an increase above 60 ms of QTc interval from baseline identifies patients at very high risk of developing malignant arrhythmias. In these patients, it would be extremely useful to pursuit a continued ECG monitoring, even outside the intensive care unit, in order to detect and promptly treat dangerous arrhythmias. However, it is also important to reassess at least on a daily basis the QTc interval in these patients, since relevant modifications should be discussed in a dedicated team that includes a cardiologist, in order to evaluate the risk/benefit ratio of the therapy. COVID-19 is a complex disease which should be managed by a multidisciplinary team especially in patients with multiple conditions.^35^ The cooperation among different specialists is essential for determining the best therapeutic strategy and clinical management. It is also important to prevent complications due to drug interactions, side effects and underestimation of potential risks. Figure 2 suggests a quick and simple algorithm that might help both cardiologists and non-cardiologists in the management of the arrhythmic risk before and during the treatment with the specific drugs used against SARS-CoV2.

This figure shows a *schematic algorithm* that can be easily followed in order to evaluate the precipitating factors of QTc interval prolongation when COVID-19 treatment should be started. The algorithm stresses the importance of a dedicated team that should discuss, together with a cardiologist, if the risk outweighs the benefit of the treatment.

**Main messages**

- Drug interaction raises concerns about arrhythmic burden in patients with COVID-19.
- COVID-19 therapies and antiarrhythmic drugs should be carefully used together.
- Daily QTc interval monitoring in patients at high risk of developing arrhythmias.
- The importance of a COVID team that includes a cardiologist.

**Key references**

1. Chan KW, Wong VT, Tang SCW. COVID-19: an update on the epidemiological, clinical, preventive and therapeutic evidence and guidelines of Integrative Chinese-Western Medicine for the management of 2019 novel coronavirus disease. Am J Chin Med. 2020 M
2. Clerkin KJ, Fried JA, Raikhelkar J, Sayer G, Griffin JM, Masoumi A, Jain SS, Burkhoff D, Kumaraiah D, Rabbani L, Schwartz A, Uriel N. Coronavirus disease 2019 (COVID-19) and cardiovascular disease. Circulation. 21 March 2020.
3. Driggin E, Madhavan MV, Bikdeli B, Chuich T, Laracy J, Bondi-Zoccai G, Brown TS, Nigoghossian C, Zidar DA, Haythe J, Brodie D, Beckman JA, Kirtane AJ, Stone GW, Krumholz HM, Parikh SA. Cardiovascular considerations for patients, healthcare workers, and health systems during the coronavirus disease 2019 (COVID-19) Pandemic. J Am Coll Cardiol. 18 March 2020.
4. Huang C, Wang Y, Li X, Ren L, Zhao J, Hu Y, *et al* Clinical features of patients infected with 2019 novel coronavirus in Wuhan, China. Lancet 24 January 2020.
5. The Liverpool Drug Interaction Group. http://www.covid19-druginteractions.org/

**Current research questions**

- Which are the exact mechanisms upon which arrhythmias develop in patients with COVID-19?
- Why are arrhythmias relatively common manifestations in patients affected by COVID-19?
- Does SARS-CoV2 directly affect the cardiac conduction system?

**Multiple choice questions**

1. The onset of arrhythmias in patients affected by COVID-19 is:
  - Attributable to multiple factors.
  - Mainly caused by hypoxia.
  - Attributable to scar tissue.
2. The most common cause for arrhythmias in COVID-19 patients is:
  - Drug interaction.
  - Scar tissue.
  - Direct myocardial injury.
3. Patients are identified as patients at very high risk of developing arrhythmias if:
  - Baseline QTc interval is above 500 ms.
  - QTc interval increases above 60 ms from baseline.
  - QTc interval is below 450 ms.
4. In COVID-19 patients it is very important to:
  - Frequently assess QTc interval.
  - Consider drug interaction.
  - Avoid electrolyte imbalance.
5. Hydroxychloroquine:
  - Does not cause a QTc interval prolongation.
  - May be responsible for arrhythmias onset.
  - Should be use with caution if other pro-arrhythmic drugs are used.

**Answers**

(A) True (B) False (C) False
(A) True (B) False (C) False
(A) True (B) True (C) False
(A) True (B) True (C) True
(A) False (B) True (C) True
